# Complete Resolution of Tumor Burden of Primary Cardiac Non-Hodgkin's Lymphoma

**DOI:** 10.1155/2016/2124975

**Published:** 2016-12-22

**Authors:** Rina Mauricio, Ofole Mgbako, Adam Buntaine, Andre Moreira, Albert Jung

**Affiliations:** Departments of Medicine and Pathology, New York University School of Medicine, New York City, NY, USA

## Abstract

Primary cardiac tumors are a rare set of benign and malignant neoplasms found in the heart or pericardium. We describe a patient presenting with nonspecific symptoms and ultimately diagnosed with primary cardiac non-Hodgkin's lymphoma (PCL). Our patient had extensive tumor in the right ventricle, which extended into the right atrium and right ventricular outflow tract. The tumor also encased the right coronary artery, which manifested as ischemic changes on EKG and cardiac MRI. The patient was treated with chemotherapy and achieved complete remission, with dramatic and full resolution of the mass on repeat echocardiography in nine weeks. More studies are needed to understand the optimal management and prognosis of patients with PCL.

## 1. Case Presentation

A 78-year-old female with a medical history of hypertension presented to the emergency department with fever and shortness of breath. Three weeks prior, the patient had presented to her primary care physician with fever to 102.0°F and dyspnea on exertion. A chest X-ray demonstrated a left lower lobe infiltrate, and she was diagnosed with community-acquired pneumonia. She was prescribed 7 days of levofloxacin as an outpatient. Despite antibiotic treatment, her symptoms worsened and progressed to lower extremity edema, night sweats, anorexia, and nausea. During this interval, she denied chest pain, cough, orthopnea, or paroxysmal nocturnal dyspnea. Given these persistent and novel symptoms, she presented to our hospital for further evaluation and management.

On initial evaluation, she was febrile to 101.3°F, tachycardic, tachypneic, and hypoxic to 90% on room air. Physical exam showed decreased breath sounds bilaterally on her pulmonary exam and trace lower extremity edema. Her cardiac exam was normal other than tachycardia. Laboratory evaluation, including white-cell count and lactate level, was normal. Blood cultures were obtained, and she was started on empiric antibiotic therapy with intravenous vancomycin, cefepime, and azithromycin. The patient's initial presentation was thought to be progression of her pneumonia secondary to inadequate outpatient treatment.

A computed tomography scan with iodinated contrast of the chest was performed. It showed a large low to intermediate density (30–50 HU) right ventricular mass extending into the right atrium and main pulmonary outflow tract which encased the right coronary artery. Bilateral pleural effusions, compressive atelectasis, and a 25 × 32 mm prevascular lymph node in the mediastinum were also present. There was no evidence of pulmonary embolism. Lower extremity Doppler ultrasound examination was negative for venous thromboembolism. Computed tomography of the abdomen was significant for a 32 × 22 mm left adrenal mass and a 26 × 23 mm para-aortic lymph node. Brain imaging was unremarkable.

**Figure 1:**
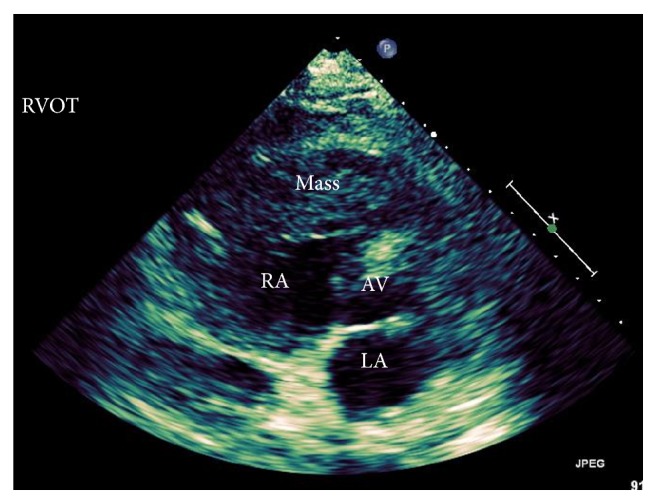
Technically difficult, parasternal short axis view at the aortic valve level showing a large heterogeneous mass occupying a majority of the RVOT.

**Figure 2:**
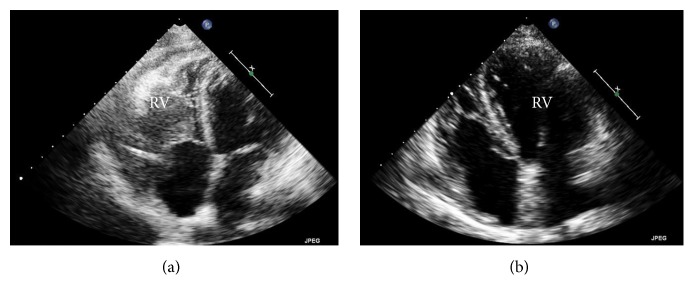
Pre- and posttransthoracic echocardiogram images. (a) Apical four-chamber view demonstrating a large mass in the right ventricle with extension into the right atrium. (b) Apical four-chamber view demonstrating complete resolution of the mass after chemotherapy.

**Figure 3:**
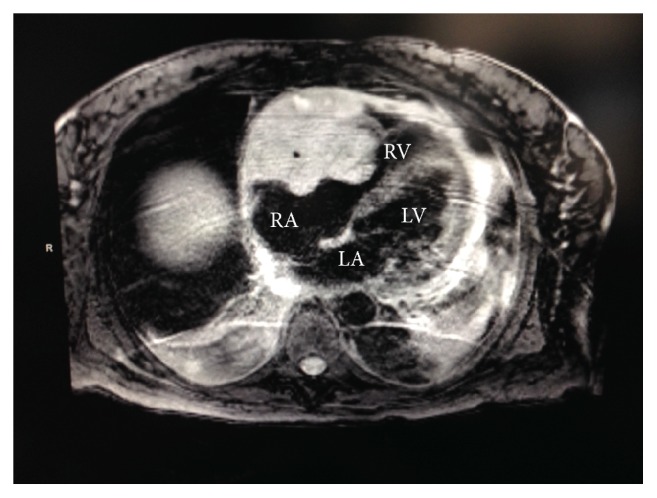
Cardiac MRI image showing a large lobulated heterogenous mass in the right ventricle which limited the opening of the tricuspid valve.

**Figure 4:**
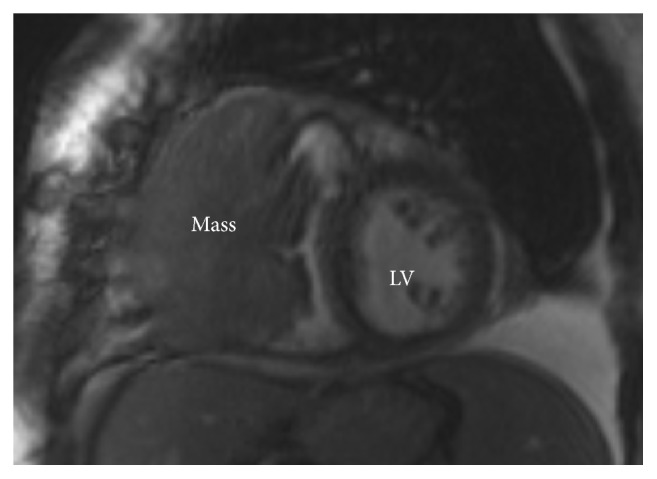
Cardiac MRI image showing a parasternal short axis view at the level of the papillary muscles demonstrates a large mass in the RV.

The right ventricular mass was presumed to be the cause of the patient's presentation and further characterization of the mass was needed. Echocardiography showed a large lobular mass that filled most of the right ventricle, extending both proximally and distally into the right atrium and right ventricular outflow tract. The mass appeared adherent to the right atrium and ventricle and created both near-complete right ventricular cavity obliteration and significant obstruction of the right ventricular outflow tract with a peak gradient of 27 mmHg (normal RVOT gradient < 4 mmHg) (Figures 1 and 2). A cardiac MRI confirmed a large, lobulated, heterogeneous right ventricular mass measuring 57 × 79 × 97 mm (in AP, transverse, and craniocaudal dimensions), causing limited opening of the tricuspid valve. The mass was predominantly isointense on T1-weighted images and hyperintense on T2-weighted images. Its imaging characteristics were most concerning for invasive malignancy (Figures 3 and 4).

**Figure 5:**
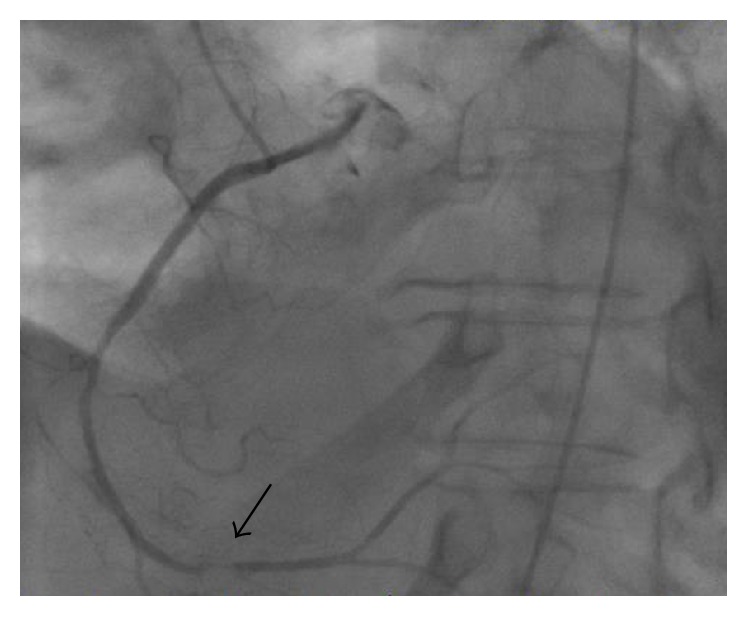
Cardiac catheterization image of the right coronary artery demonstrating a significant filling defect in the distal artery.

Interestingly, the presenting and subsequent EKGs showed normal sinus rhythm but with signs of ischemia in the inferior wall. The patient denied any symptoms of chest pain during and preceding this presentation. Right and left heart catheterizations were performed to biopsy the mass and assess for coronary artery disease that could explain the evidence of inferior wall ischemia on EKG. The left heart catheterization demonstrated severe stenosis of the distal right coronary artery due to encasement from the cardiac mass, which correlated to the territory of distal infarction (Figure 5). Interestingly, the normal translational movement of the right coronary artery with each systole was absent, likely, due to fixative compression of the mass. Additionally, cardiac MRI showed late gadolinium enhancement in the territory of the inferior wall.

**Figure 6:**
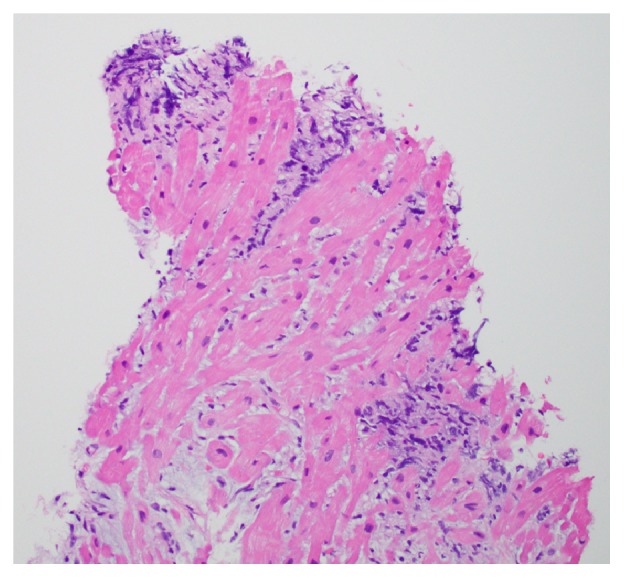
Pathology specimen of cardiac tissue demonstrating lymphocytes infiltrating the cardiac muscle, consistent with cardiac lymphoma.

**Figure 7:**
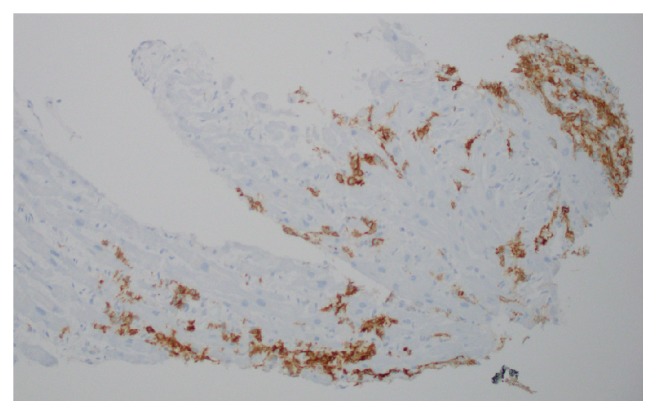
Immunohistochemical stain of cardiac muscle showing that the cells are positive for CD791, a B-cell marker.

Endomyocardial biopsy of the mass was performed. Pathology showed an aberrant population of CD5(−) and CD10(−) large B-lymphocytes compatible with diffuse large B-cell lymphoma (Figures 6 and 7). Cytology from pleural fluid corroborated this finding.

The patient was diagnosed with primary cardiac non-Hodgkin's lymphoma. The extent of her tumor burden was impressive, as was its external compression of the right coronary artery leading to evidence of ischemia on both EKG and cardiac MRI.

The patient was transferred to the oncology service and started on reduced dose R-CHOP (rituximab max rate of 300 mg/hour, cyclophosphamide 500 mg/m^2^, doxorubicin hydrochloride 35 mg/m^2^, vincristine sulfate 1.4 mg/m^2^ with max 2 mg, and prednisone 100 mg daily for 5 days) chemotherapy and closely monitored for tumor lysis syndrome. After one course of chemotherapy, the patient was discharged with a plan for continued chemotherapy as an outpatient.

She subsequently received 7 cycles of chemotherapy. Remarkably, transthoracic echocardiography and PET/CT performed 9 weeks after her initial diagnosis of primary cardiac lymphoma revealed complete resolution of the right ventricular mass, pleural effusions, as well as adrenal nodule, and mediastinal and para-aortic lymph nodes (Figure 2).

## 2. Discussion

Primary cardiac tumors are rare, with the incidence ranging from 0.0017% to 0.33% based on eight large autopsy series [1]. Most primary cardiac tumors are benign, making up 58–77% of all primary cardiac tumors. Atrial myxoma is the most common benign primary cardiac tumor (24–37%). Malignant tumors of the heart are less common and comprise 24–42% of all primary cardiac tumors. Angiosarcoma is the most common malignant primary cardiac tumor, representing 7-8% of all primary cardiac tumors [1]. The incidence of cardiac involvement in metastatic lymphoma ranges from 7.5 to 9.3% in one case series [2]. In contrast, the incidence of primary cardiac lymphoma is exceedingly rare, ranging from 0.04% to 1.8% of all primary cardiac tumors [1].

Primary cardiac lymphoma (PCL) is defined as non-Hodgkin's lymphoma exclusively or mainly located in the heart or pericardium [3]. PCL is primarily found in the right side of the heart, the most frequent location being the right atrium [4, 5]. Most patients present with nonspecific symptoms such as dyspnea, edema, chest pain, or palpitations [4–6]. Studies have suggested that the diagnosis of PCL should be considered in patients presenting with a cardiac mass or unexplained, refractory pericardial effusion, and heart failure [4]. Additionally, PCL has been associated with arrhythmias, heart block, or angina. In our case, the EKG showed evidence of inferior ischemia, which was likely due to external compression of the distal RCA by the tumor.

Multimodality cardiac imaging studies are a cornerstone of the diagnosis of PCL helping to assess the location, size, and character of the cardiac mass. While cardiac tumors are often first identified on transthoracic echocardiography, transesophageal echocardiogram is superior to transthoracic echocardiogram (TTE) in identifying cardiac lesions, identifying 92% versus 55% of lesions in one study. This advantage is primarily due to its ability to better characterize lesions in the left atrium, left atrial appendage, and SVC [7]. Gadolinium enhanced cardiac magnetic resonance (CMR) imaging can detect tumor location and morphology and has a >90% sensitivity in detecting cardiac lymphoma [4, 8]. The use of multiple imaging modalities, such as TTE, which best demonstrates the functional impact of a cardiac mass, as well as CMR for sizing, location, and tumor characterization is likely the optimal approach in the diagnosis of cardiac lymphoma [9]. Although imaging studies are important in the work-up of a cardiac mass, tissue diagnosis via endomyocardial biopsy (or other tissue biopsy) is necessary to confirm any suspected diagnosis due to the imperfect specificity of these imaging studies.

There are many different types of lymphoma that affect the heart as primary tumors. One review of all reported cases [*n* = 40] between 1995 and 2002 demonstrated that the majority of primary cardiac lymphomas are diffuse large cell (60%), with the second most common being large cell (5%), immunoblastic (5%), lymphoblastic (5%), diffuse medium-cell (5%), diffuse small-cell (5%), and diffuse (5%) [6]. In this review, specific treatment was given to 36 patients with complete remission obtained in only 15 patients. Of the 25 patients who died, average survival time was 16 weeks (range 1 to 48 weeks) [6].

Primary cardiac lymphoma is highly sensitive to chemotherapy, and so it is widely used as the primary treatment modality. In a recent review of 197 reported cases of PCL, chemotherapy was given to 89% of patients, with CHOP and subsequently R-CHOP being the most common regimens. Seventy-nine percent of treated patients with follow-up data available had a partial or complete response to therapy by any modality, with 59% achieving complete response [10]. Partial or complete surgical resection was performed in 28% of patients and radiation therapy in 20% of patients [10]. These debulking procedures are generally reserved for cases of PCL in which there is severe cardiac obstruction and/or hemodynamic compromise as a result of the mass. To our knowledge, there have been no studies comparing chemotherapy alone or chemotherapy with either surgical resection or radiation therapy. This is likely due to the rarity of the disease.

Reported complete remission rates have ranged from 38% to 71%, illustrating the limited knowledge of this disease [6, 11]. Risk factors for poor prognosis include immunocompromised state, extracardiac disease, left ventricular involvement, and the absence of arrhythmia. Extracardiac disease and left ventricle involvement are thought to represent greater burden of disease. It has also been postulated that arrhythmias lead to earlier presentation, imaging, and thus detection of disease [10]. Further studies are needed to elucidate the management and prognosis of this disease.

## 3. Conclusion

PCL is difficult to diagnose given vague symptomatology and nonspecific signs on physical exam. Patients with PCL often present with a nonresolving, unexplained respiratory complaint as did our patient. Multimodality imaging is important in guiding the work-up of cardiac masses, though it is insufficient to confirm a diagnosis. For the latter, tissue biopsy is necessary. There are no randomized clinical trials comparing the treatment regimens for primary cardiac lymphoma. These regimens are generally devised by an oncologist and directed at the type of lymphoma involved. Indeed, studies show that R-CHOP is the most commonly used chemotherapy regimen with a strong response rate. Surgical resection and radiotherapy appear to have some benefit in cases of severe obstruction, but the addition of either or both has not been proven superior to chemotherapy alone.
